# Comparative analysis of polymerization efficiency and degradation indicators of adhesive resin cements and preheated restorative composites

**DOI:** 10.1038/s41598-026-38779-y

**Published:** 2026-02-12

**Authors:** Dóra Jordáki, Katalin Böddi, Zsuzsanna Őri, Vivien Vass, Sándor Kunsági-Máté, József Szalma, Edina Lempel

**Affiliations:** 1 Department of Restorative Dentistry and Periodontology, University of Pécs Medical School, Tüzér Street 1, Pécs, 7623 Hungary; 2 Department of Biochemistry and Medical Chemistry, University of Pécs Medical School, Szigeti Street 12, Pécs, 7624 Hungary; 3 Food and Nutrition Analytical Laboratory, Institute of Basic Health Sciences and Analytical Laboratory Research, Faculty of Health Sciences, University of Pécs, Vörösmarty Street 4, Pécs, 7621 Hungary; 4 Department of Physical Chemistry and Materials Science, Institute of Chemistry, Faculty of Sciences, University of Pécs, Ifjúság Street 6, Pécs, 7624 Hungary; 5 Department of Organic and Medicinal Chemistry, Faculty of Pharmacy, University of Pécs, Honvéd Street 1, Pécs, 7624 Hungary; 6 János Szentágothai Research Center, University of Pécs, Ifjúság Street 12, Pécs, 7624 Hungary; 7 Department of Oral and Maxillofacial Surgery, University of Pécs Medical School, Tüzér Street 1, Pécs, 7623 Hungary

**Keywords:** Adhesive cement, Preheating, Degree of conversion, Monomer elution, Water sorption, Solubility, Ceramic restoration, Chemistry, Materials science

## Abstract

Preheated restorative resin-based composites (RBCs) have been suggested as alternative luting agents for ceramic restorations; however, their polymerization and long-term degradation behavior require further clarification. This study compared a preheated RBC with conventional light-cured and dual-cured adhesive resin cements regarding degree of conversion (DC%), monomer elution, water sorption (WSo), and solubility (Sol). Standardized lithium disilicate overlays were luted to ceramic abutments using preheated Estelite Σ Quick (EQ_55°C), Variolink Esthetic LC (VE_LC), or Variolink Esthetic DC (VE_DC). DC% was assessed by micro-Raman spectroscopy, monomer elution using high-performance liquid chromatography at 3, 10, and 17 days, and WSo/Sol per ISO 4049 at 30, 60, and 90 days. VE_DC showed the highest DC% (72.1%), while EQ_55°C presented the lowest (59.9%). Monomer elution was significantly higher for VE_LC and VE_DC compared to EQ_55°C and decreased over time for all groups. WSo peaked at day 60 but remained significantly lower for EQ_55°C. Sol followed the pattern VE_DC > VE_LC > EQ_55°C, decreasing progressively with time. These results indicate that the preheated RBC demonstrated reduced monomer release and lower hydrolytic degradation, supporting its potential use as a stable luting alternative for indirect ceramic restorations.

## Introduction

 Posterior ceramic restorations are widely used to overcome limitations of direct resin-based composites (RBCs)^1,2^. Their clinical success depends significantly on the properties of the luting agents used^3–5^. Resin-based cements, especially light-cured and dual-cured systems, are popular due to their handling ease, mechanical strength, and esthetic compatibility^6,7^. However, light attenuation through ceramics can impair the degree of conversion (DC) in deeper regions, potentially increasing monomer elution, which weakens the cement layer and poses cytotoxic or allergenic risks^7–11^.

Beyond elution, water sorption (WSo) and solubility (Sol) critically affect long-term stability. Water uptake disrupts filler–matrix bonds, plasticizes the resin, reduces mechanical strength, and promotes further degradation^12^. Sol reflects the loss of unreacted or hydrolytically cleaved components^13^. Elevated WSo and Sol are linked to marginal breakdown, discoloration, and bacterial colonization^2,14,15^. Adhesive resin cements, with lower filler content and more low-molecular-weight monomers, are especially prone to hydrolytic degradation^6,16,17^.

To address these concerns, preheated restorative RBCs have been proposed as alternative luting agents. Preheating reduces viscosity, enhances flow, and may improve marginal adaptation and mechanical performance due to their higher filler load and lower resin matrix volume^18–20^. Several studies confirm their ability to achieve acceptable film thickness (≤ 120 μm) and bond strength when used as cements^18,21–24^. Moreover, preheated RBCs may improve DC when curing through thicker ceramics (3–4 mm), where light transmission is significantly reduced^25^.

While the mechanical properties and bond strength of preheated RBCs have been well studied, their polymerization kinetics and degradation behavior remain underexplored, particularly in direct comparison with resin cements, despite the extensive research on DC and monomer elution in the latter^26–29^. A comprehensive evaluation of DC, monomer elution, WSo, and Sol is required to ensure reliable material comparison, particularly when polymerization is performed through ceramics with limited light transmission^30^.

The aim of this study was to evaluate and compare the DC and monomer elution of a preheated restorative RBC used as a luting agent, relative to conventional light-cured and dual-cured resin cements, using micro-Raman spectroscopy and reversed-phase high-performance liquid chromatography (RP-HPLC), respectively. Additionally, WSo and Sol were assessed. The following null hypotheses were tested: there are no differences in (1) DC, (2) monomer elution, (3) WSo, and (4) Sol among the investigated materials.

## Methods

Three resin-based materials suitable for bonding ceramic restorations were tested during the study, a light-cure adhesive resin cement Variolink Esthetic LC (VE_LC) and a dual-cure adhesive resin cement Variolink Esthetic DC (VE_DC) (Ivoclar Vivadent). Furthermore, a highly filled restorative RBC, Estelite Ʃ Quick (Tokuyama Dental) that had been preheated to 55 °C prior to use was also tested (EQ_55°C). The chemical composition of materials is presented in Table 1.

**Table 1:** Materials, manufacturers, classification, and composition of the investigated adhesive resin cements and preheated resin-based composites.

Material (code) (shade)	Type	Resin system; Initiator	Filler	Filler load
Variolink esthetic LC (VE_LC) (Light)	Light-curing adhesive resin cement	UDMA, 1,10-DDDMA; GDMA, Ivocerin	0.04–0.2 μm ytterbium trifluoride and spheroid mixed oxide	38 vol% 64 wt%
Variolink esthetic DC (VE_DC) (Light)	Dual-curing adhesive resin cement	UDMA, 1,10-DDDMA; GDMA Ivocerin, hydrogen-peroxide/thiocarbamide redox system	0.04–0.2 μm ytterbium trifluoride and spheroid mixed oxide	38 vol% 64 wt%
Estelite sigma Quick (EQ_55°C) (A1 Enamel)	Conventional submicron RBC preheated to 55 °C	BisGMA, TEGDMA; CQ, RAP	0.1–0.3 μm monodispersing spherical silica-zirconia filler; prepolymerized filler of silica-zirconia and copolymer	71 vol% 82 wt%

RBC: resin-based composite; BisGMA: bisphenol-A diglycidil ether dimethacrylate; UDMA: urethane dimethacrylate; TEGDMA: triethylene glycol dimethacrylate; 1,10-DDDMA: 1,10-decandiol dimethacrylate; GDMA: glycerin-1,3-dimethacrylate; CQ: Camphoroquinone; RAP: Radical Amplified Photopolymerization; LC, light-cure; DC, dual-cure; vol%, volumetric %; wt%, weight%.

To avoid variability associated with natural tooth substrates, each RBC was used to lute a prefabricated ceramic overlay onto a ceramic abutment model simulating a prepared tooth. The use of standardized ceramic abutments ensured consistent dimensions and uniform cement space, thereby eliminating anatomical and compositional differences inherent to natural dentin, which could otherwise influence water sorption, monomer release, and solubility. High-translucency lithium disilicate overlays (GC Initial LiSi Block; shade A2; GC Europe) were fabricated using computer-aided design (CAD) and computer-aided manufacturing (CAM) techniques. The abutments used for cementation were likewise milled from the same lithium disilicate ceramic material but in a low-translucency formulation, in order to simulate the optical and structural characteristics of a prepared tooth substrate while maintaining standardized geometry. Milling procedures were carried out according to the manufacturer’s instructions.

**Fig. 1:**
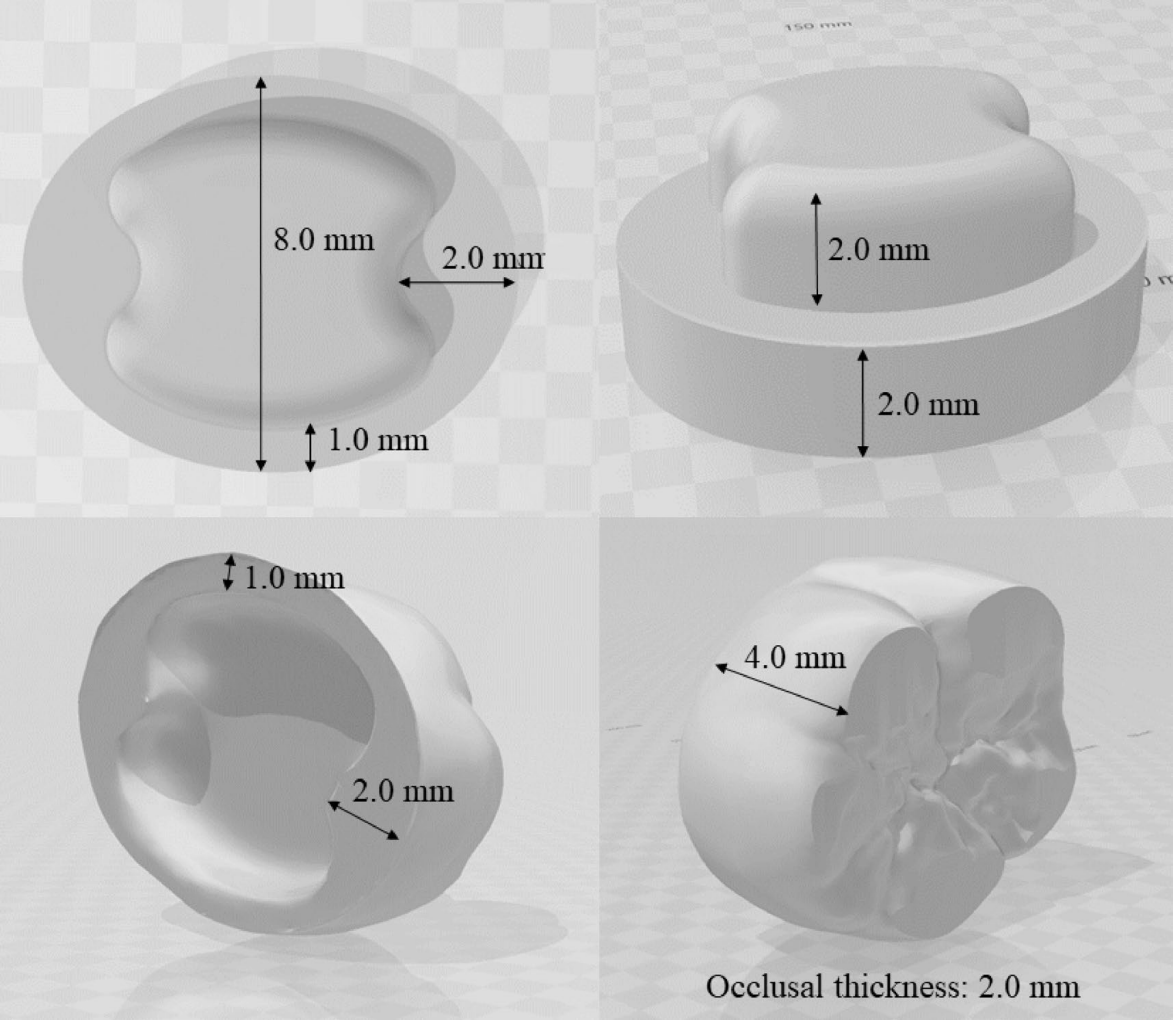
Three-dimensional digital designs of the lithium disilicate ceramic abutment (base) and overlay, created using Autodesk Fusion software (Autodesk Inc., San Francisco, CA, USA). The images illustrate the structural dimensions from different planes, including occlusal thickness and axial wall height.

Figure 1 shows the digital plan (Autodesk Fusion, Autodesk Inc.) of the ceramic overlay and abutment from different planes, indicating their respective dimensions. The occlusal thickness of the overlays was set to 2 mm, as this value is generally recommended to ensure adequate fracture resistance of lithium disilicate restorations in functional load-bearing areas. A digital caliper (Digital Caliper, Mitutoyo) with an accuracy of 0.001 mm was used to determine the final dimensions of each ceramic sample.

The two ceramic components (overlay and abutment) were bonded using the three tested materials (*n* = 3 × 10). Prior luting the ceramic components had been pretreated with 9% buffered hydrofluoric acid (Porcelain Etch, Ultradent Products) for 20 s, followed by the application of a silane coupling agent (Silane, Ultradent Products) and left to dry for one minute. The adhesive cements were mixed with an Automix syringe. The single-dose restorative RBC was preheated to 55 °C for 15 min using an RBC warmer (Ena Heat, Micerium). RBC temperature was measured with a non-contact infrared thermometer (TESTO 845, Testo), resolution 0.1/1°C; sampling rate: 10 measurements/second. Ceramic overlays were preheated in the warming device to reduce heat loss during cementation. A standard amount of luting agent was applied to the intaglio surface of each overlay. Based on a previous study, applying a manual seating force of 5–10 N during placement of the ceramic restorations resulted in a reproducible luting layer thickness of approximately 100 ± 10 μm^31^. Overlays were seated on the abutments under a controlled constant manual load using the Optrasculpt instrument (Ivoclar Vivadent), while the magnitude of the applied force was verified with a handheld algometer (Force Dial FDK 16; Wagner, Greenwich, USA) to ensure consistency. Excess cement was removed with a microbrush before light curing. Polymerization was carried out from three corresponding directions (analogous to the occlusal, buccal, and lingual aspects) for 20 s each using a third-generation LED curing unit (Bluephase PowerCure, Ivoclar Vivadent) in high mode (1180 mW/cm² average irradiance; 385–515 nm spectral range). The output of the light-curing unit was monitored regularly to ensure consistent irradiance throughout the polymerization procedures using a radiometer (Bluephase Meter, Ivoclar Vivadent). The margins were finished and polished using the Sof-Lex (3 M, ESPE) pop-on extra thin disc kit.

Five specimens from each group (*n* = 3 × 5) were bisected longitudinally along the mesiodistal axis. The 72-hour post-cure DC values were evaluated using a confocal Raman spectrometer (Labram HR 800, HORIBA Jobin Yvon S.A.S.). Spectra were recorded at the center of the luting layer (10 measurements per point, 10 s integration), then averaged. A 20 mW He-Ne laser (λ = 632.817 nm) and 10× optical magnification (Olympus) were used. The spectral resolution (~ 2.5 cm⁻¹) allowed distinction between the 1639 and 1609 cm⁻¹ peaks. Non-polymerized RBC spectra served as reference. Data analysis was performed in LabSpec 5.0 using eight-order polynomial fitting and baseline subtraction. The DC% was calculated (Eq. 1) based on the ratio of aliphatic to aromatic carbon double bonds before and after curing using the following equation:

1 $$\:\mathrm{D}\mathrm{C}{\%}=\left[1-\left(\frac{\mathrm{R}2}{\mathrm{R}1}\right)\right]\mathrm{x}100$$

Equation 1: Degree of conversion (DC%) was calculated using the ratio of peak intensities at 1639 cm⁻¹ (R1 as unpolymerized C = C) and 1609 cm⁻¹ (R2 as polymerized C = C) in RBCs.

Five bonded ceramic samples per adhesive tested (*n* = 3 × 5) were immersed in 1.0 mL of 75% ethanol/water in glass vials and incubated at 37 °C for 3 days, then transferred to fresh medium for two additional 7-day periods. Storage solutions were collected after 3, 10, and 17 days to analyze eluted monomers using RP-HPLC. Measurements followed previously published conditions, including identical instrumentation and mobile phase, except for a 5-minute increase in separation time with a constant 95% acetonitrile eluent^32^. Although experimental settings remained unchanged, different monomers were analyzed, resulting in distinct retention times. All analyses were conducted under consistent conditions to ensure comparability. Standard solutions of analyzed molecules were used to identify target compounds in the eluates based on their retention times: UDMA (15.257 min), BisGMA (17.540 min), TEGDMA (10.320 min), EDMAB (13.477 min), DDDMA (32.023 min), GDMA (6.610 min), CQ (9.180 min), and Ivocerin (22.627 min). Table 1 lists the full names of the compounds analyzed. All peaks were well resolved. Monomer concentrations in RBC eluates were quantified using calibration curves based on peak area and expressed per 1 mg of RBC.

In accordance with ISO 4049:2019, five disc-shaped specimens (15 ± 1 mm diameter, 1 ± 0.1 mm thickness) were prepared per material using a custom mold. Final dimensions were verified with a digital caliper (± 0.001 mm; ABS Digimatic). Volume (V, mm³) and surface area (mm²) were calculated based on measured values (Eq. 2):

2 $$\:\mathrm{V}={\uppi\:}{\mathrm{r}}^{2}\mathrm{t}$$

Equation 2: Volume calculation formula (where π = 3.14; r is the radius of cross section; and t is the thickness of the specimen).

Specimens were first conditioned in a desiccator at 37 ± 2 °C for 22 h, then transferred to a second desiccator at 23 ± 1 °C for 2 h. They were weighed using an analytical balance (± 0.01 mg; Balance XPR205, Mettler Toledo), and the drying-weighing cycle was repeated until mass stabilized (m₁; change ≤ 0.1 mg/24 h). Each specimen was immersed in 10 mL of distilled water in sealed glass vials at 37 °C. Mass measurements (m_2_) were taken after 1 min of blotting at the following time points: 1, 3 h; days 1–7, 14, 21, 30, 32, 33, 37, 44, 51, 60, 62, 63, 67, 74, 81, and 90. After weighing, samples were returned to their respective vials. The water was refreshed weekly, maintaining a consistent volume of 10 mL.

After the sorption phase, specimens were transferred to a desiccator and weighed at intervals of 1, 2, 3, 4, 5, 6, 7, 14, 21, 30, 32, 33, 37, 44, 51, 60, 62, 63, 67, 74, 81, and 90 days. Drying continued until mass change between two consecutive 24-hour measurements was ≤ 0.1 mg, at which point the final constant mass (m₃) was recorded. Water sorption (WSo) and solubility (Sol) were calculated using the following formulas:

3 $$\:\mathrm{W}\mathrm{S}\mathrm{o}=\left[\frac{{\mathrm{m}}_{2}{-\mathrm{m}}_{3}}{\mathrm{V}}\right]$$

Equation 3: Water sorption calculation formula (m₂: mass after 90-day immersion; m₃: mass after desorption; V: specimen volume). WSo represents the residual water content within the specimen post-immersion, which cannot be entirely removed through reconditioning.

4 $$\:\mathrm{S}\mathrm{o}\mathrm{l}=\left[\frac{{\mathrm{m}}_{1}{-\mathrm{m}}_{3}}{\mathrm{V}}\right]$$

Equation 4: Solubility calculation formula (m₁: conditioned mass before immersion; m₃: mass after desorption; V: volume). Sol reflects the amount of material lost from the RBC during water storage.

Sample size was calculated using a standard formula (Eq. 5) [33] based on prior studies on monomer elution and DC%.^34,35^

5 $$\:\mathrm{n}=\frac{{({\mathrm{z}}_{1-\frac{{\upalpha\:}}{2}}+{\mathrm{z}}_{1-{\upbeta\:}})}^{2}{({\mathrm{s}}_{1}+{\mathrm{s}}_{2})}^{2}}{({\mathrm{M}}_{1}-{{\mathrm{M}}_{2})}^{2}}$$

Equation 5: Sample size formula, where z = standard score; α = probability of Type I error at 95% confidence level = 0.05; z_1−α/2_ = 1.96 for 95% confidence; β = probability of Type II error = 0.20; 1 − β = the power of the test = 0.80; z_1−β_ = value of standard normal variate corresponding to 0.80 value of power = 0.84; s_1_, s_2_ =standard deviations; M_1_, M_2_ = group means.

For monomer elution s₁ = 0.011, s₂ = 0.003; *M₁* = 0.22, M₂ = 0.19; the calculated sample size is 1.7/group. For DC% s₁ = 1.5, s₂ = 0.8; M₁ = 67.4, M₂ = 64.5; the calculated sample size is 4.9/group. Based on these estimates and methodological consistency, five specimens per group (*n* = 5) were deemed sufficient.

Statistical analyses were conducted in SPSS (v26.0; IBM). Mean and standard deviations were calculated for monomer elution, DC%, WSo, and Sol. Normality was assessed using the Kolmogorov–Smirnov test. Group differences were evaluated using one-way ANOVA with Tukey’s post hoc test. Repeated measures ANOVA was used to compare WSo and Sol after 30, 60, and 90 days. A general linear model (GLM) assessed effect sizes for variables such as material type, curing mode, and time. Pearson’s correlation coefficient was calculated to explore associations between parameters. P-values < 0.05 were considered statistically significant.

## Results

**Fig. 2:**
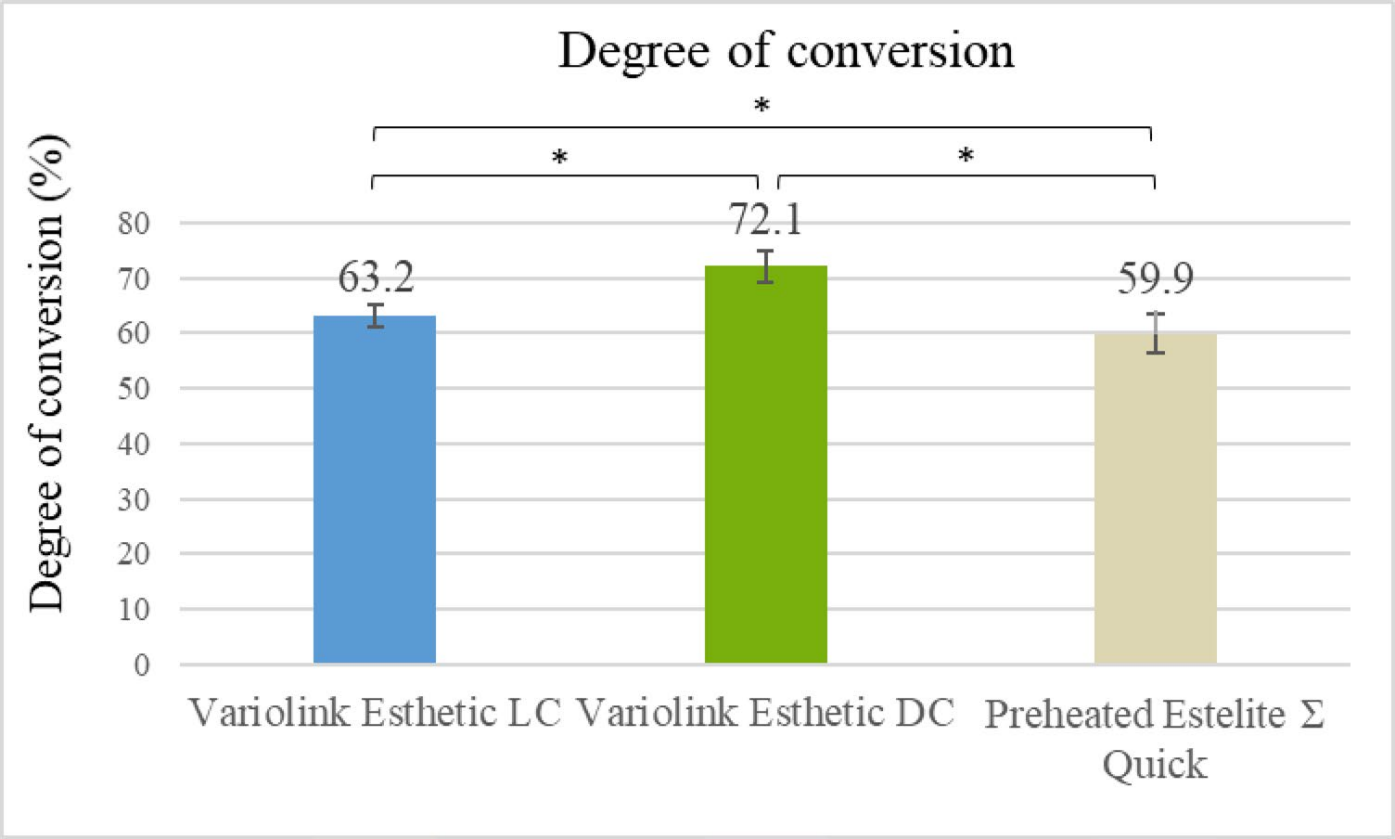
Degree of conversion of VE_LC, VE_DC, and preheated EQ_55°C measured by micro-Raman spectroscopy through a 2 mm thick ceramic disc. Asterisks (*) indicate statistically significant differences (*p* < 0.05; one-way ANOVA, Tukey’s post hoc tests).

DC% values differed significantly among the tested materials (*P* < 0.05). VE_DC showed the highest conversion (72.1%), while EQ_55°C had the lowest (59.9%) (Fig. 2). GLM analysis confirmed a strong, statistically significant effect of material type [F(2,12) = 55.2, partial eta squared (pη²) = 0.90, *P* < 0.001] and curing mode [F(1,12) = 79.1, pη² = 0.87, *P* < 0.001] on DC%.

**Fig. 3:**
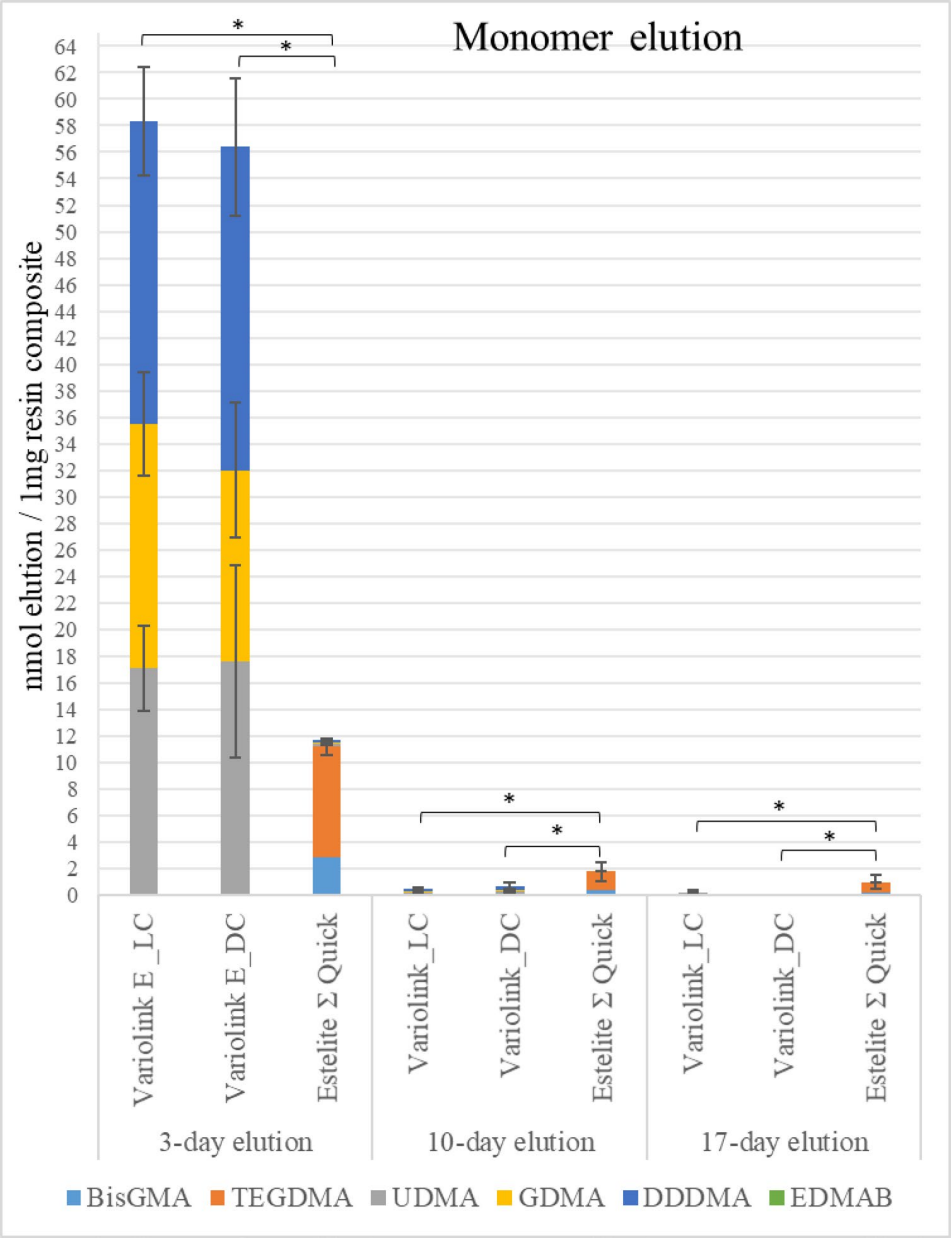
High-performance liquid chromatography (HPLC) analysis of monomer elution from VE_LC, VE_DC, and preheated EQ_55°C after 3-, 10-, and 17-day immersion periods in distilled water at 37 °C. Values are expressed in nmol eluted per mg of resin composite. Asterisks (*) indicate statistically significant differences according to ANOVA and Tukey’s post hoc test (*p* < 0.05). (Abbreviations: UDMA, urethane-dimethacrylate; BisGMA, bisphenol A-glycidyl methacrylate; TEGDMA, triethylene glycol dimethacrylate; DDDMA, 1,10-decandiol dimethacrylate; GDMA, glycerin-1,3-dimethacrylate, EDMAB, ethyl 4-dimethylaminobenzoate.

**Fig. 4:**
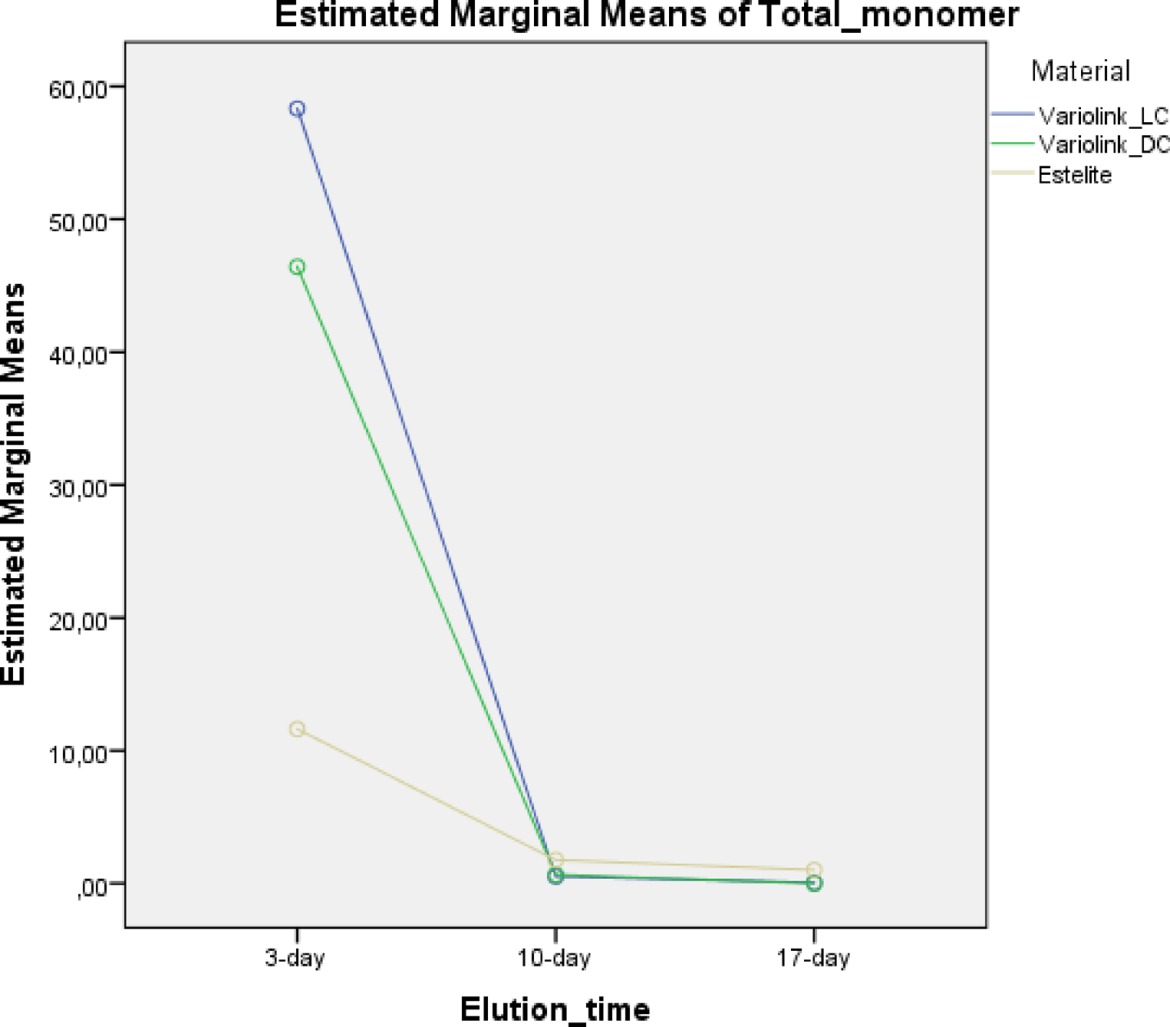
Estimated marginal means of total monomer elution (nmol elution/1 mg resin-based material) from the tested materials during the detection time intervals (3, 10, and 7 days). The values were calculated based on repeated measures general linear model analysis.

Monomer elution differed significantly among materials and over time (Figs. 3 and 4). After 3 days, VE_LC and VE_DC showed comparable profiles, both releasing significantly more monomers than EQ_55°C (*P* < 0.05). UDMA, DDDMA, and GDMA were the dominant eluates in resin cements, even though BisGMA was not listed in manufacturer data. In contrast, EQ_55°C primarily released TEGDMA and BisGMA. No release of photo- or co-initiators was detected. Total monomer release decreased over time across all materials.

Elution on days 10 and 17 was significantly lower than on day 3 (*P* < 0.05). EQ_55°C exhibited residual release and differed significantly from the adhesive cements (*P* < 0.05). GLM revealed significant effects of material type [F(2,36) = 24.94, *P* < 0.001, pη² = 0.58] and elution time [F(2,36) = 204.76, *P* < 0.001, pη² = 0.92] on total monomer release, indicating large effect sizes. In contrast, curing mode had no significant impact [F(1,13) = 0.75, *P* = 0.40, pη² = 0.05]. Pearson correlation showed a moderate positive relationship on day 3 (*r* = 0.60, *P* = 0.02), suggesting higher DC% was associated with slightly increased initial elution. At later time points, strong negative correlations were found (Day 10: *r* = − 0.68, *P* = 0.005; Day 17: *r* = − 0.75, *P* = 0.001). All correlations were statistically significant, highlighting the dynamic relationship between polymerization and elution over time.

**Fig. 5:**
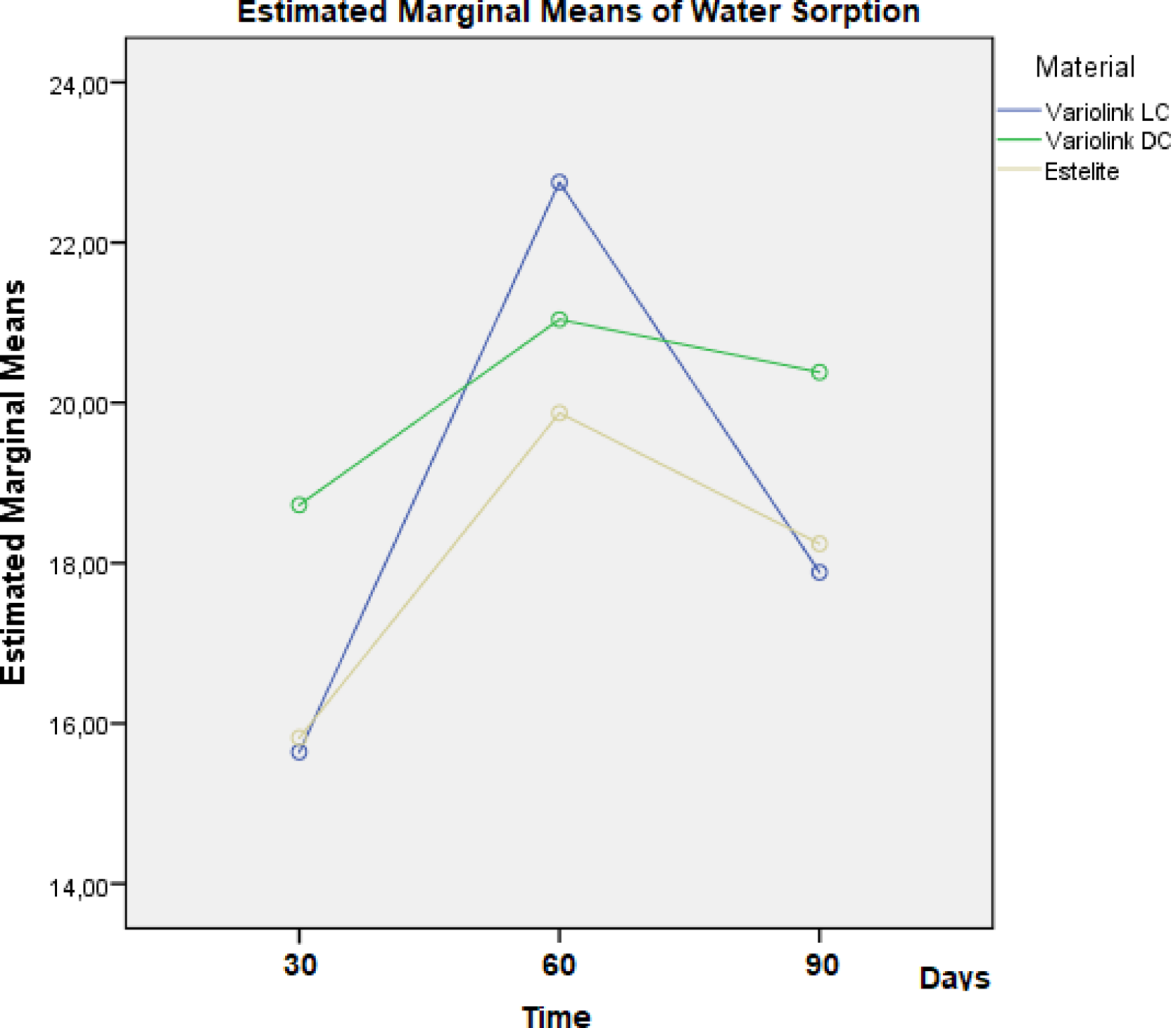
Estimated marginal means of water sorption (µg/mm³) across all sampling time points for the three tested resin-based composites. The values were calculated based on repeated measures general linear model analysis.

WSo differed significantly among materials at days 30 and 90, but not at day 60 (Table 2). At Day 30 VE_DC showed significantly higher WSo than VE_LC (*P* = 0.004) and EQ_55°C (*P* = 0.007); no difference between VE_LC and EQ_55°C (*P* = 0.971) was observed. At Day 60 differences were not statistically significant [F(2,12) = 0.53, *P* = 0.60]. At Day 90 VE_DC again showed significantly higher WSo (*P* < 0.01) than both other materials. WSo increased in all groups until day 60, then decreased—most notably in VE_LC. EQ_55°C consistently showed the lowest WSo values (Fig. 5). GLM analysis showed a significant effect of time F(2,19.82) = 23.86, *P* < 0.001, pη² = 0.47], but not of material (*P* = 0.165), material × time interaction [*P* = 0.415] or curing mode (*P* = 0.075).

**Table 2:** Mean water sorption and solubility values (g/mm³) of the tested materials at 30, 60, and 90 days.

	Material	Day 30 (Mean ± SD)	Day 60 (Mean ± SD)	Day 90 (Mean ± SD)
Water sorption	Variolink Esthetic DC	18.73 ± 1.35	21.04 ± 1.07	20.38 ± 0.86
	Variolink Esthetic LC	15.64 ± 0.63	22.75 ± 7.53	17.89 ± 1.01
	Estelite Ʃ Quick	15.82 ± 1.47	19.87 ± 1.02	18.24 ± 0.47
Solubility	Variolink Esthetic DC	14.85 ± 3.37	8.82 ± 2.21	3.95 ± 1.35
	Variolink Esthetic LC	8.31 ± 3.08	6.11 ± 0.70	2.22 ± 0.51
	Estelite Ʃ Quick	7.01 ± 1.92	4.25 ± 0.50	1.50 ± 0.92

Pearson correlation analysis revealed a statistically significant positive relationship between DC% and WSo at both day 30 (*r* = 0.67, *P* = 0.007) and day 90 (*r* = 0.65, *P* = 0.008), indicating that higher DC% were associated with increased WSo. However, no significant correlation was found at day 60 (*r* = 0.08, *P* = 0.771). No significant correlations were found between WSo and monomer elution, although a moderate, non-significant negative correlation was seen at day 17 vs. day 30 WSo (*r* = − 0.45, *P* = 0.095).

**Fig. 6:**
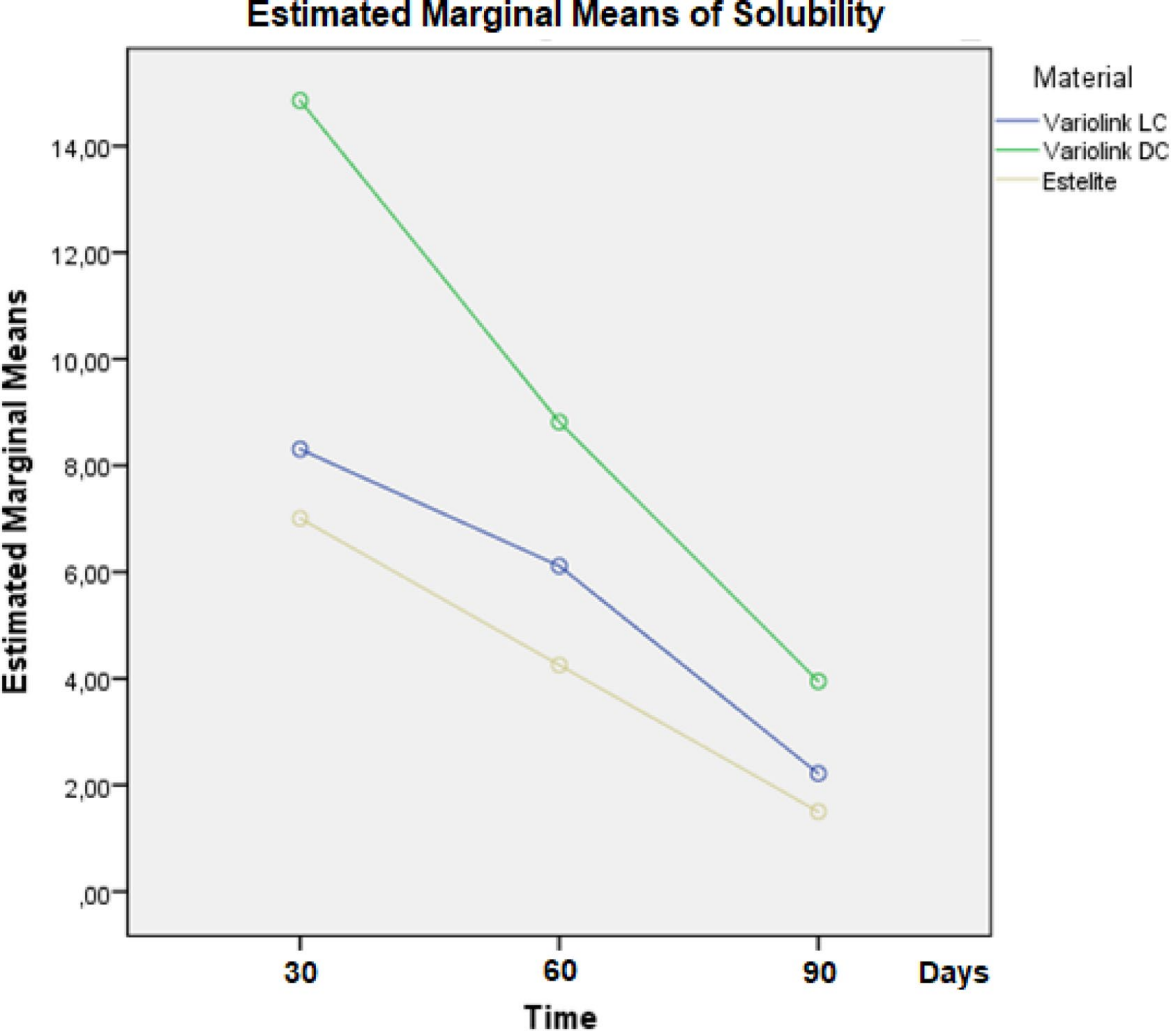
Estimated marginal means of solubility (µg/mm³) measured at three time points (30, 60, and 90 days) for VE_LC and VE_DC adhesive cements, and EQ_55°C preheated restorative resin-based composite.

Across all time points, VE_DC exhibited the highest Sol values, EQ_55°C the lowest (Table 2). One-way ANOVA confirmed statistically significant differences in Sol among the tested materials at all three time points: day 30 [F(2,12) = 10.80, *P* = 0.002], day 60 [F(2,12) = 13.98, *P* = 0.001], and day 90 [F(2,12) = 8.06, *P* = 0.006]. VE_DC had significantly higher Sol than both VE_LC (*P* = 0.002) and EQ_55°C (*P* < 0.001). No significant difference between VE_LC and EQ_55°C (*P* = 0.62). The trend remained consistent over time. Repeated measures ANOVA confirmed a significant effect of time [F(2,24) = 67.8, *P* < 0.001, ηp² = 0.78], material [*P* < 0.001, ηp² = 0.77], and curing mode [*P* < 0.001, ηp² = 0.73], but no significant time × material interaction (*P* = 0.415) (Fig. 6).

Pearson correlation analysis revealed statistically significant positive associations between DC% and Sol at all time points. A strong correlation was found at day 60 (*r* = 0.81, *P* < 0.001), followed by day 30 (*r* = 0.75, *P* = 0.001), and a moderate correlation at day 90 (*r* = 0.59, *P* = 0.020). Correlation analysis between monomer elution and Sol (measured at day 30) revealed no statistically significant associations. A weak positive correlation was observed with day 3 elution (*r* = 0.34, *P* = 0.217), while day 10 showed a weak negative correlation (*r* = − 0.37, *P* = 0.179). A moderate negative correlation was identified with day 17 elution (*r* = − 0.51, *P* = 0.054), which approached but did not reach statistical significance. Lack of elution data beyond day 17 limited long-term correlation analysis.

## Discussion

This study evaluated three resin-based luting materials—VE_DC, VE_LC, and preheated EQ_55°C—with regard to DC%, WSo, Sol, and monomer elution. Each material was used to lute a lithium disilicate overlay onto a standardized ceramic abutment model simulating a prepared tooth. The use of uniform ceramic abutments ensured consistent geometry and cement space, eliminating substrate-related variability that could otherwise influence degradation-related outcomes. This model also reproduces a key clinical condition in which the thickness and translucency of ceramic restorations attenuate curing light, thereby potentially reducing DC and affecting the corresponding material properties^30,36^.

A clear material-dependent trend was observed: VE_DC showed the highest WSo, Sol, and monomer elution, followed by VE_LC, while EQ_55°C consistently exhibited the lowest degradation. In contrast, the DC% displayed an inverse trend, with VE_DC achieving the highest conversion and EQ_55°C the lowest. These findings led to the rejection of all null hypotheses initially proposed. Statistically significant differences were observed among the materials for each of the investigated parameters. The results support and expand upon previous research, emphasizing that the chemical stability and hydrophilic behavior of RBCs are strongly influenced by their composition over time. Despite their higher DC%, conventional adhesive resin cements demonstrated a more pronounced hydrophilic character compared to the preheated restorative RBC. A DC% in RBCs is widely associated with improved physical and chemical properties^37^. During polymerization, an extensive crosslinking has been demonstrated to reduce the amount of unreacted monomers and enhance resistance to water sorption and solubility^38,39^. This correlation is well-established in dental research, demonstrating that a more complete cure results in a more stable and less permeable material^12,40^. However, this relationship is not universally applicable, as it is strongly dependent on the material’s composition and the specific polymerization conditions. Although higher DC% is generally expected to correlate with reduced WSo, Sol, and monomer release, our findings diverged from this trend. VE_DC showed the highest conversion but also the greatest degradation, suggesting that DC% alone does not fully predict material stability. Other factors—such as free volume, hydrophilicity, filler–matrix interactions, and curing mode—also influence resistance to degradation^17,41,42^. Adhesive resin cements generally have a higher monomer content than restorative RBCs—for example, VE_LC and VE_DC contain 62% monomers—enabling higher polymerization, as reflected in the elevated DC% observed^43–45^.

Beyond quantity, monomer type also affects conversion. Reactive, low-viscosity monomers such as UDMA and GDMA improve mobility and crosslinking, enhancing polymerization^46^. VE_DC and VE_LC use UDMA, 1,10-DDDMA, and GDMA, while EQ_55°C is based on Bis-GMA and TEGDMA. UDMA’s flexibility and lower viscosity support higher DC%, and 1,10-DDDMA acts as a flexible spacer promoting network formation^42,47,48^. In contrast, Bis-GMA’s rigidity hinders mobility, limiting conversion, while TEGDMA, though promoting conversion, increases shrinkage and heterogeneity^49^. The synergistic use of UDMA, 1,10-DDDMA, and GDMA in VE provides an optimal balance of flexibility and crosslinking potential, resulting in more complete polymerization^50^.

Despite the identical filler type (0.1 μm spherical silica), VE_LC and VE_DC have significantly lower filler content (38 vol%) than EQ_55°C (71 vol%), contributing to their higher DC% (63.2% and 72.1% vs. 59.9%). High filler loading can limit polymerization by reducing monomer mobility and scattering light^51^. Although EQ_55°C was preheated to 55 °C, a clinically relevant temperature for ceramic cementation that has been demonstrated to reduce film thickness and enhance monomer flow and polymerization^19^, the lower filler load and more reactive monomer matrix in VE materials still resulted in higher DC%. VE_DC and VE_LC share the same composition, but VE_DC achieved a significantly higher DC% due to its dual-cure initiator system (Ivocerin + redox initiator), which enhances conversion under reduced light transmission, aligning with previous findings^52–54^. EQ_55°C uses a camphorquinone/amine system with radical amplification photoinitiator (RAP) to extend working time and reduce stress. Despite its high filler load and reduced translucency, EQ_55°C still reached a clinically acceptable DC% (59.9%) under the same curing conditions, though statistically lower than the others. As the materials being tested differed in their photoinitiator systems (camphorquinone versus camphorquinone + Ivocerin), a polywave light-curing unit was used to ensure adequate spectral coverage and standardized polymerization under ceramic restorations; therefore, the reduced DC% of EQ_55°C is more likely attributable to light attenuation by dense filler content through scattering and absorption^55^. Notably, most resin cements achieve DC% values around 60% in clinical conditions^29^.

A notable finding was the positive correlation between DC% and monomer elution in adhesive cements. Within the first 3 days post-polymerization, both LC and DC cements released approximately five times more monomers than the preheated RBC—amounts comparable to those eluted from five to ten medium-sized restorations^56^. This can be attributed to the higher monomer content in cements, which results in more residual free monomers after curing^57^. Although elution from cements dropped by ~ 99–100% within 1–2 weeks, the preheated RBC showed lower total elution but more persistent release—approximately 85% reduction after the initial peak, with detectable amounts still present at day 17^58^. These findings suggest that monomer elution is not solely governed by DC%, but also by factors such as network architecture, filler content, and diffusion pathways. Similar elution kinetics have been observed in other resin systems, where rapid initial release is followed by slower, prolonged diffusion due to saturation of the cross-linked matrix^59–61^. The use of 75% ethanol as the extraction medium was intended to simulate intraoral chemical conditions^62^. Ethanol-based solvents prolong elution compared to water by swelling the polymer matrix and facilitating deeper monomer diffusion^57^.

The literature consistently supports a positive correlation between monomer elution and Sol, as higher residual monomer content leads to greater leaching and mass loss^41^. Materials with lower filler loading also tend to absorb more water and dissolve more readily^42^. WSo further promotes degradation via hydrolysis and plasticization, enhancing monomer release and structural breakdown^17,63^. In this study, the DC adhesive cement showed higher WSo and Sol than the LC variant at most time points. This may stem from the heterogeneous network formation typical of dual-cure systems, where chemically cured regions—especially in deeper or less translucent areas—exhibit lower cross-link density and increased porosity^41,64^. Although auto-mixing reduces air entrapment compared to manual methods, dual-cure formulations may still present greater porosity than light-cure resins^29^. Increased porosity facilitates capillary fluid uptake, which can lead to hydrolytic degradation, filler–matrix debonding, microleakage, discoloration, and compromised mechanical^65^. The chemical composition of resin cements—especially their hydrophilic monomer content—strongly influences degradation behavior. While higher DC% is generally associated with lower WSo and Sol, surface energy (hydrophilicity) shows an even stronger correlation with degradation^42,66^. The elevated WSo, Sol, and elution observed in VE_DC likely reflect its more hydrophilic matrix and potential for network defects. In contrast, EQ_55°C showed lower degradation, likely due to its hydrophobic, highly filled, and cross-linked structure, which enhances matrix–filler interactions and network density^67^. Interestingly, its relatively delayed monomer release at days 10 and 17 may indicate that free monomers in the dense network elute more slowly^68,69^. Although DC% reflects curing efficiency, it alone does not determine hydrolytic stability. Material factors—such as monomer composition, network uniformity, and filler–matrix interactions—play a greater role. The findings emphasize that, despite its high DC%, VE_DC exhibited greater degradation, while EQ_55°C’s formulation demonstrated superior moisture resistance.

Limitations of the study include the single time-point measurement of DC% (72-hour ), although prior research confirms that methacrylate resins show minimal conversion changes after 24 h^70^. Standardized ceramic abutments were used to reduce variability, though they may not fully replicate natural dentin properties. Although pre-heated RBCs has been shown to improve flowability and handling characteristics, maintaining the elevated temperature throughout the cementation procedure may be challenging under clinical conditions. It has been demonstrated that RBCs undergo a rapid loss of heat immediately following their removal from the warming device. This phenomenon can result in a substantial increase in viscosity prior to the complete seating of the restoration. This temperature drop has the potential to affect various parameters, including film thickness, adaptation, and polymerization behavior^19,71^. Consequently, the results obtained under controlled in vitro conditions may not fully reflect the clinical situation, where temperature control is more difficult to standardize. Despite the meticulous standardization of film thickness in the present study, employing a controlled seating force to yield a reproducible cement layer of approximately 100 ± 10 μm, minor variations may still occur. Such variations have the potential to affect light transmission and the DC, particularly in the context of ceramic restorations. Consequently, this factor must be taken into account when extrapolating the present in vitro findings to clinical conditions. Additionally, no adhesive was applied to isolate cement elution; however, this excludes the adhesive’s potential contribution to total monomer release—a factor shown to increase cytotoxicity^72^.

## Conclusions

Within the limitations of this study, the following conclusions can be drawn:

1. The dual-cure resin cement (VE_DC) achieved the highest DC% but also showed the greatest early monomer elution.
2. The light-cure variant (VE_LC) had slightly lower DC% but similar elution, indicating that high conversion does not necessarily limit monomer release.
3. The preheated restorative RBC (EQ_55°C) exhibited the lowest overall elution despite lower DC%, suggesting a more stable polymer network.
4. WSo and Sol values showed material-dependent trends over time, with EQ_55°C demonstrating the most favorable degradation profile.

From a clinical perspective, preheated restorative RBCs may serve as a viable alternative to resin cements in certain indirect restorations. Their enhanced chemical stability and acceptable polymerization suggest potential for improved long-term outcomes. However, further studies in clinically relevant settings are needed to validate these findings.

## Data Availability

The datasets generated during and/or analyzed during the current study are available from the corresponding author on reasonable request.
